# Manipulation of immunodominant variable epitopes of norovirus capsid protein elicited cross-blocking antibodies to different GII.4 variants despite the low potency of the polyclonal sera

**DOI:** 10.1128/jvi.00611-25

**Published:** 2025-05-30

**Authors:** Kentaro Tohma, Lauren A. Ford-Siltz, Joseph A. Kendra, Gabriel I. Parra

**Affiliations:** 1 Division of Viral Products, Center for Biologics Evaluation and Research, Food and Drug Administration 333555 , Silver Spring, Maryland, USA; University of Michigan Medical School, Ann Arbor, Michigan, USA

**Keywords:** noroviruses, calicivirus, vaccine, antigen resurfacing, cross-neutralizing antibody, diarrhea

## Abstract

Norovirus is a major cause of viral acute gastroenteritis. The predominant genotype, GII.4, presents a continuous emergence of new variants that could escape immunity developed in previous infections. The emergence of GII.4 variants is associated with multiple mutations on five antigenic sites (A, C, D, E, and G) of the major viral capsid protein. Antigenic site A includes most immunodominant epitopes, while antigenic site G shifted its immunodominance during the evolution of GII.4 noroviruses. This interrelation of immunodominance and high variability challenges the development of universal vaccines for GII.4 noroviruses. In this study, we designed an immunogen that aims to elicit antibodies toward conserved regions by mutating epitopes from antigenic sites A and G. Immunization of mice with mutant virus-like particles (VLPs) in which antigenic sites A and G were resurfaced with alanine mutations (12∆AG) elicited multiple cross-blockade monoclonal antibodies against different GII.4 variants. However, the carbohydrate-blockade titers at the serum level were low as compared with wild-type VLPs as the result of low frequency and/or low potency of these cross-blockade antibodies. This study confirmed that cross-reactive antibodies can be elicited by mutating highly variable epitopes, but further studies are required to overcome the low immunogenicity of these conserved epitopes for the development of vaccine candidates that could elicit broadly protective responses.

**IMPORTANCE:**

The fast-evolving nature of RNA viruses is a major obstacle for vaccine design and development. Protective antibodies are often directed to highly variable sites, so viruses could rapidly escape from immunity acquired from previous infections or vaccination. Here, we designed mutant norovirus virus-like particles (VLPs) in which the immunodominant and highly variable epitopes were resurfaced into alanine for refocusing immune responses toward conserved epitopes. The mutant VLPs could elicit multiple broadly cross-reactive antibodies against different GII.4 noroviruses that emerged in the 1970s–2010s; however, the frequency of these cross-reactive antibodies was low at the serum level. While further investigations are required to enhance the potency of these cross-reactive responses, this study opens a new avenue for the rational development of efficacious norovirus vaccines.

## INTRODUCTION

**Fig 1:**
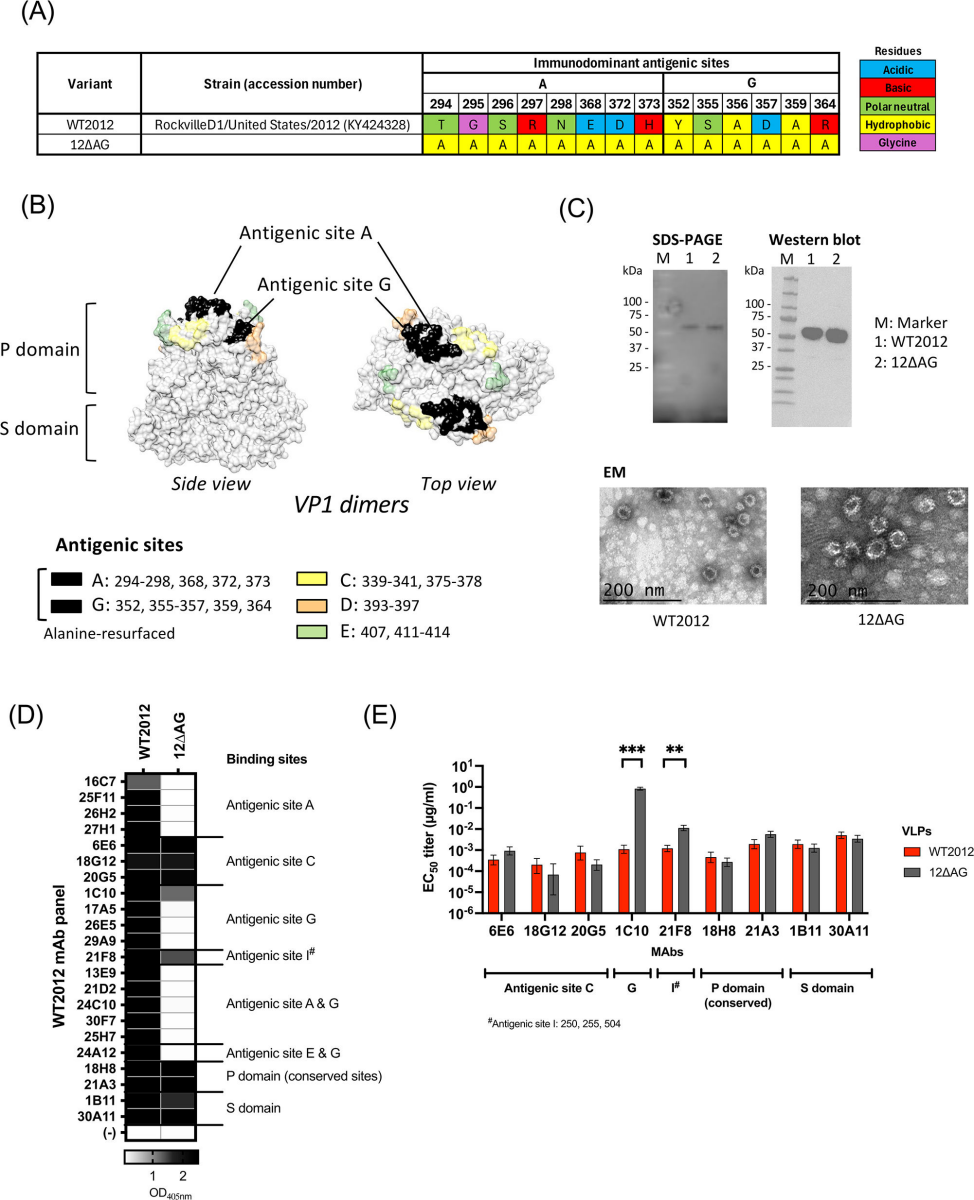
Development of 12∆AG VLPs. (**A**) Sequence of antigenic sites A and G of wild-type Sydney 2012 variant strain, WT2012 (SY), and 12∆AG where the residues on both antigenic sites were mutated to alanine. The colors on the cell indicate the chemical properties of the amino acid. (**B**) The three-dimensional structure of the VP1 protein of GII.4 norovirus (PDB ID: 7K6V). Antigenic sites that are involved in viral neutralization and HBGA blockade, including those found mutated in this study (sites A and G), were indicated. (**C**) The pictures indicate SDS-PAGE gel (top left), Western blot (top right), and the electron microscopy images (bottom) of purified WT2012 (SY) and 12∆AG VLPs. Norovirus VP1-specific cross-reactive mAb, 30A11 (22), was used to confirm the expression of the VP1 protein in the Western blot analysis. (**D**) ELISA data to confirm the binding (or not binding) of previously characterized WT2012 (SY) mAbs (18) to 12∆AG VLPs. The heatmap shows OD_405nm_ values in ELISA, using 10 µg/mL of purified mAbs and 0.5 µg/mL of VLPs. (**E**) MAbs that bind to 12∆AG VLPs were further tested for binding by 10-fold serial dilution of mAbs. The bar graph shows the difference of EC_50_ titers between WT2012 (SY) and 12∆AG VLPs. The error bars represent the 95% confidence intervals of estimates in nonlinear regression analyses. **P < 0.01; ***P < 0.001 in *t*-test.

Norovirus is a major cause of viral acute gastroenteritis in all ages. In healthy individuals, symptoms resolve within days, but severe and chronic disease can develop in vulnerable populations, such as malnourished children, the elderly, or immunocompromised individuals. Indeed, norovirus has been associated with up to 200,000 deaths annually worldwide (1), yet no vaccines or specific treatments are currently available. A major barrier to developing effective vaccines against norovirus is that reinfections can occur throughout life (2–5). Reinfections are associated with decrease of protective immunity (6, 7) and the presence of diverse norovirus genotypes (8). Based on the sequence diversity, over 40 different genotypes have been reported infecting humans. In addition to the diversity presented at the genotype level, the predominant genotype, GII.4, presents a continuous emergence of new variants that can escape the immunity developed by previous infections (9–13). These variants have been defined as groups of viruses that are phylogenetically distinct from each other and have been detected to be causing infections in at least two distant locations (14). Since the 1990s, multiple variants (e.g., Grimsby 1995 [or US95_96], Farmington Hills 2002, Hunter 2004, Yerseke 2006a, Den Haag 2006b, New Orleans 2009, and Sydney 2012) have been associated with large and concurrent outbreaks worldwide. Other variants have also been reported (Sakai 2003 [or Asia 2003], Osaka 2007, Apeldoorn 2007, San Francisco 2017, and Hong Kong 2019); however, despite presenting antigenic differences and circulating in different countries (15, 16), these variants did not cause large outbreaks worldwide. The emergence of these GII.4 variants was demonstrated to be linked to the acquisition of multiple mutations mapping to five antigenic sites (namely, A, C, D, E, and G, see Fig. 1) on the capsid of the virus (9, 12). This viral capsid is formed by the major capsid protein, viral protein 1 (VP1), which is structurally divided into the shell (S) and protruding (P) domains. The latter is further subdivided into the relatively conserved P1 domain and highly variable P2 domain (17). These five antigenic sites map to the P2 subdomain and are involved in eliciting antibodies that can neutralize the virus and block the interaction between the capsid protein and histo-blood group antigen (HBGA) carbohydrates (18), which are cell attachment factors that facilitate infection (19–21).

After a rapid turnover of epidemic variants during 2002–2012, the Sydney 2012 variant has been circulating as the most predominant norovirus for over a decade without major antigenic changes (23). Previous studies demonstrated that antigenic site A includes most immunodominant B cell epitopes among GII.4 variants (9, 18), while antigenic site G presented changes in its immunodominance during the evolution of GII.4 noroviruses (18). Both antigenic sites, particularly antigenic site A, present multiple mutations among the different GII.4 variants (12), which constitutes a roadblock for the development of vaccines against different and, potentially, future emerging GII.4 variants. Most responses directed to conserved regions of the capsid protein, S and P1, have been shown to be cross-reactive (22, 24–27), yet most of these antibodies are neither neutralizing nor present HBGA-blockade activity (18, 22, 26). Notably, cross-blocking and neutralizing antibodies have been detected and demonstrated to map to the conserved P1 or P1/P2 interface of VP1 (18, 28, 29), suggesting that cross-protective vaccines can be produced. In this study, we designed an immunogen that would refocus the immune responses toward conserved regions of VP1 with the goal of eliciting cross-neutralizing responses to different GII.4 variants.

## RESULTS

Because the antigenic sites A, C, D, E, and G are the ones eliciting most neutralizing antibody responses (18), we first designed and expressed a VP1, with all the residues on these five antigenic sites replaced with alanine. The expression of the protein was confirmed by Western blot, but this mutant VP1 did not form VLPs to be aggregated in CsCl gradient ultracentrifugation, suggesting that an excess of mutations at these antigenic sites prevented the assembly of the VLPs (data not shown). Thus, we next developed a mutant VP1, 12∆AG, in which all 14 residues of the two most immunodominant antigenic sites A and G on the currently circulating Sydney 2012 variant [WT2012 (SY)] virus were mutated to alanine (Fig. 1A and B). The 12∆AG mutant VP1 was successfully expressed, formed VLPs (Fig. 1C), and showed reactivity with monoclonal antibodies (mAbs) mapping to linear and conformational epitopes of the wild-type WT2012 (SY) VLPs (18) (Fig. 1D). As expected, mAbs mapping to antigenic sites A and G did not show reactivity with 12∆AG VLPs. An exception was mAb 1C10, which mapped to antigenic site G, but still bound to the 12∆AG VLPs at a weaker level (>500-fold difference of EC_50_ titers, *P* = 0.0008 in t test; Fig. 1E and ELISA OD_405nm_ curves presented in Fig. S1). Antigenic site G of WT2012 (SY) presents two alanine residues (Fig. 1A), suggesting that residues 356 and/or 359 are anchor residues for 1C10 binding. Importantly, the 12∆AG VLPs bound to mAbs mapping to epitopes outside the mutated areas at a similar level with WT2012 VLPs; three blockade/neutralizing mAbs mapping to antigenic site C (6E6, 18G12, 20G5), 1 mAb mapping to antigenic site I (21F8), two cross-blockade/neutralizing mAbs mapping to the conserved sites of the P domain (18H8 and 21A3) (18), and two cross-reactive mAbs mapping to the S domain (Fig. 1E). The mAb 21F8 presented weaker binding to the mutant VLPs (*P* = 0.0017 in t test); however, the reduction of binding (increase of EC_50_ titers) was subtle, with the difference of the EC_50_ titer <10 fold. Together, these data demonstrate that 12∆AG mutant VLPs still present conformational epitopes that are involved in HBGA blockade and viral neutralization outside the antigenic sites A and G.

**Fig 2:**
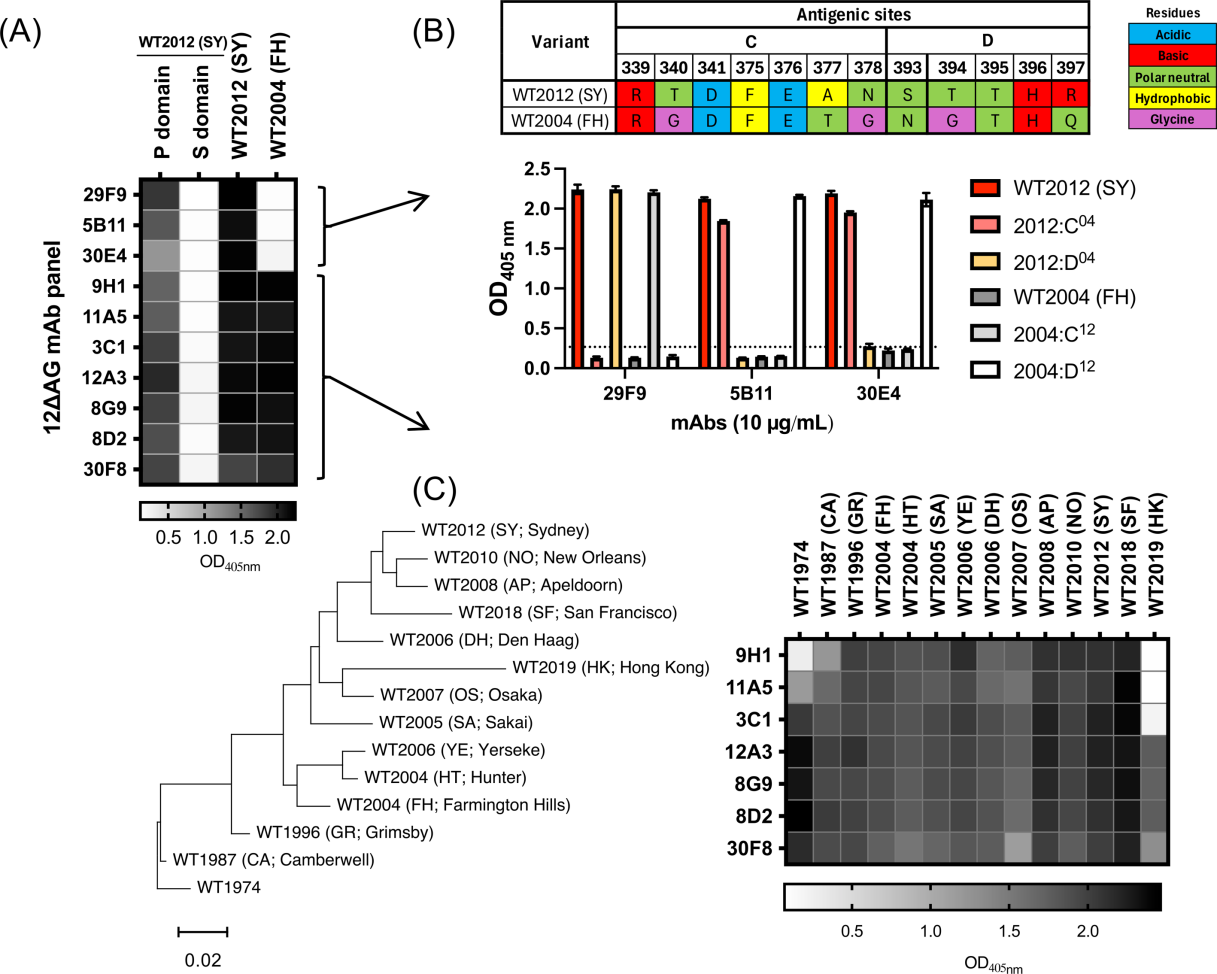
Characterization of mAbs produced from mice immunized with 12∆AG VLPs. (**A**) Ten mAbs were newly developed after immunization of 12∆AG VLPs to mice. They were selected based on their binding patterns to P and S domains of WT2012 (SY) and homologous and heterologous reactivities to the VLPs of WT2012 (SY) and WT2004 (FH) viruses. The heatmap shows OD_405nm_ values in ELISA, using 10 µg/mL of purified mAbs and 0.5 µg/mL of proteins or VLPs. (**B**) Three WT2012 (SY)-specific mAbs were characterized using antigenic sites C- or D-swapped mutants, in which residues on individual antigenic sites were swapped between WT2012 (SY) and WT2004 (FH) viruses (18). Sequences of antigenic sites C and D of WT2012 (SY) and WT2004 (FH) are shown on the top panel. The bar graph on the bottom indicates ELISA OD_405nm_ values, using 10 µg/mL of purified mAbs and 0.5 µg/mL of VLPs. The dashed line indicates the cutoff value calculated using OD_405nm_ values taken from negative control wells (mean + 2 × standard deviation). (**C**) Breadth of the cross-reactivity from seven mAbs was confirmed using VLPs from viruses detected from 1974 to 2019. The heatmap shows OD_405nm_ values in ELISA, using 10 µg/mL of mAbs and 0.5 µg/mL of VLPs. The genetic relationship of the different GII.4 viruses tested is indicated in the phylogenetic tree at the left of the heatmap.

**Fig 3:**
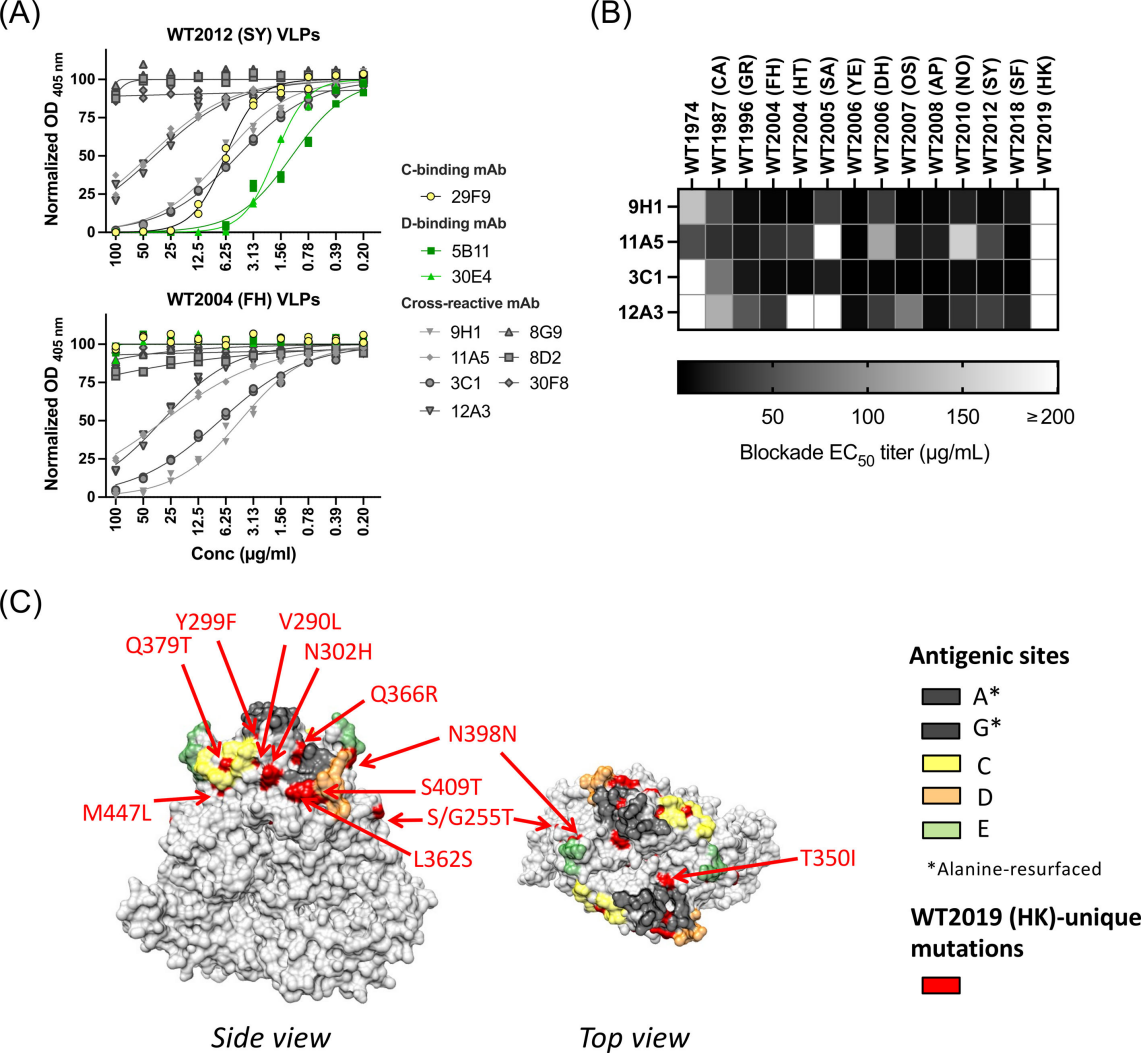
Blockade potential of cross-reactive mAbs produced from mice immunized with 12∆AG VLPs. (**A**) The homologous and heterologous HBGA-blockade antibodies were measured using blockade assays using PGM III as a source of carbohydrates. The line graphs indicate best-fit linear regression curves and normalized OD_405nm_ values in duplicate wells at twofold dilution of mAbs. (**B**) The heatmap summarizes the blockade EC_50_ titers of four cross-reactive mAbs against historical GII.4 variants that emerged between 1974 and 2019. (**C**) The three-dimensional structure of the VP1 dimers of GII.4 norovirus (PDB ID: 7K6V) indicates the position of mutations that were detected only in the Hong Kong 2019 variant in red. Position of antigenic sites is indicated by the same color palette used in Fig. 1A.

The newly developed 12∆AG VLPs were used to immunize mice with the goal of enhancing cross-neutralizing responses. First, to profile the antibodies elicited by immunization of this mutant VP1, we isolated mAbs from two mice immunized with 12∆AG VLPs. After three immunizations, 34 parental hybridoma clones positive to the immunogen (12∆AG VLPs) by ELISA were isolated. We selected 10 clones that reacted with the WT2012 (SY) VLPs and mapped to the P domain for further propagation and characterization (Fig. 2A). This selection was based on previous findings that the S domain seems to play a minimal role in viral neutralization (18, 30). All of the newly developed mAbs recognized conformational epitopes, as indicated by negative signals in the Western blot. Three of these mAbs did not bind to one of the ancestral variants, Farmington Hills 2002 [WT2004 (FH)] VLPs and were mapped to antigenic sites C (mAb 29F9) and D (mAbs 5B11 and 30E4) using antigenic site-swapped mutant VLPs (Fig. 2B). The remaining seven P-domain-mapping mAbs presented broad reactivity to GII.4 variants that emerged between 1974 and 2019 (Fig. 2C). Six of these mAbs reacted with one of the oldest GII.4 viruses detected, WT1974 (CHDC5191/United States/1974), and four mAbs reacted with the recently detected GII.4 variant, Hong Kong 2019 [WT2019 (HK)] (31). Meanwhile, the recently described variant, San Francisco 2017 [WT2018 (SF)] (32), reacted with all seven P-domain-mapping mAbs. We then tested the ability of the P-domain-mapping mAbs to block the interaction of HBGA and VLPs from homologous WT2012 (SY) and heterologous WT2004 (FH) viruses (Fig. 3A). Four cross-reactive mAbs (9H1, 11A5, 3C1, and 12A3) presented HBGA-blockade activity against both homologous and heterologous VLPs, with two mAbs (9H1 and 3C1) presenting stronger cross-blockade titers (EC_50_ = 4.353 and 7.241 µg/mL, respectively). The mAbs mapping to antigenic sites C and D presented strong to modest homologous HBGA-blockade titers (EC_50_ = 1.180–6.291 µg/mL), but no heterologous HBGA-blockade activity. We further tested the breadth of the HBGA-blockade activity of the four cross-blockade mAbs by testing VLPs representing major and minor variants that emerged from 1974 to 2019 (Fig. 3B, HBGA-blockade OD_405nm_ curves are presented in Fig. S2). Although all four mAbs cross-blocked nearly all GII.4 variants with varying potency, none were able to block HBGA binding of the Hong Kong 2019 variant. Notably, the Hong Kong 2019 variant presents multiple unique mutations on the P domain (13); thus, the binding of these cross-blockade antibodies could be affected by one (or more) of these mutations. These mutations, including 12 on the surface and 2 inside the protein, are located at antigenic site I (residue 255) and adjacent to antigenic sites A, C, D, E, and G (Fig. 3C). These residues are highly conserved among different GII.4 variants, and there are no mutations shared with Hong Kong 2019 and ancestral, WT1974, WT2004 (HT), and/or WT2005 (SA) viruses that could explain the lack of HBGA blocking by these mAbs.

**Fig 4:**
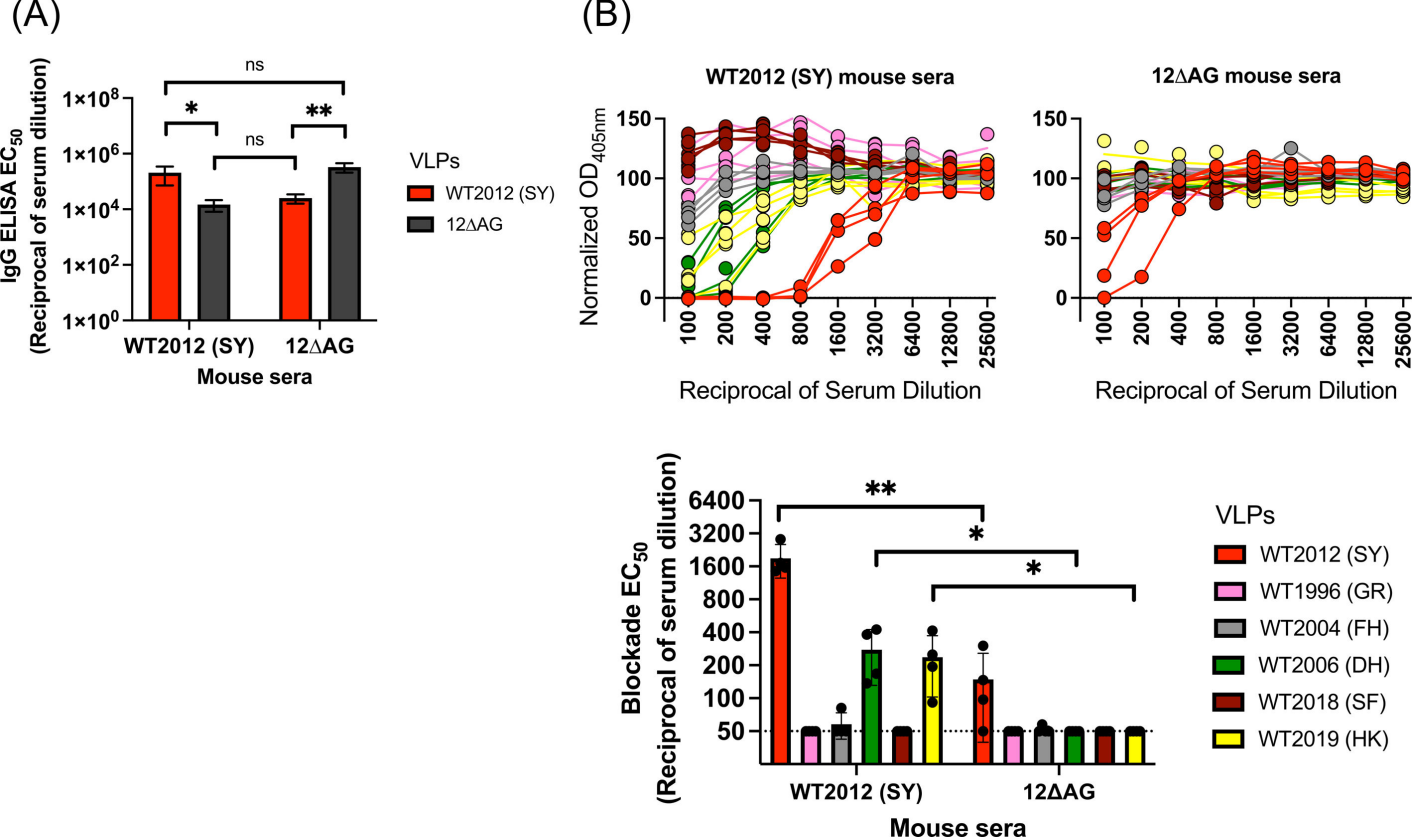
HBGA-blockade assays of sera from mice immunized with wild-type WT2012 (SY) or the mutant 12∆AG VLPs. (**A**) ELISA to measure IgG titers in WT2012 (SY)- and 12∆AG-immunized mouse sera against immunogens—WT2012 (SY) and 12∆AG mutant VLPs. The IgG titers were summarized in a bar graph using EC_50_ values. *P < 0.05; **P < 0.01 in One-way ANOVA and Šídák multiple comparison test. (**B**) The HBGA-blockade antibodies in the wild-type VLP-immunized (left) and 12∆AG VLP-immunized mouse sera (right) were measured against homologous WT2012 (SY) VLPs and heterologous VLPs including WT1996 (GR), WT2004 (FH), WT2006 (DH), WT2018 (SF), and WT2019 (HK), using blockade assays using PGM III as a source of carbohydrates. The top line graphs show normalized OD_405nm_ values from four mice in duplicate wells at twofold dilution of serum. The bottom bar graph summarizes the EC_50_ titers of individual sera against different GII.4 variants in the blockade assays. The error bars represent the 95% confidence intervals of estimates in nonlinear regression analyses. The dot indicates EC_50_ titers of four mice. The horizontal dashed line indicates the limit of detection in the blockade assays (EC_50_ = 50). *P < 0.05; **P < 0.01 in *t-*test.

We next measured blockade antibodies in serum from animals immunized with 12∆AG VLPs. Notably, despite similar serum IgG titers elicited upon immunization (Fig. 4A) and the positive results with mAbs (Fig. 3), the overall antibody responses measured using mouse hyperimmune serum indicated minimal blockade activity (Fig. 4B). While sera developed against 12∆AG VLPs still presented WT2012 (SY) homologous blockade antibodies (EC_50_ = 157.91, average of four mouse sera), the titer was significantly lower compared to sera developed against the WT2012 (SY) VLPs (EC_50_ = 1891, average of four mouse sera, *P* = 0.01102 in *t*-test, Fig. 4B). The heterologous blockade titers of anti-12∆AG mouse sera measured against variants emerged in previous and recent years, including WT1996 (GR), WT2004 (FH), WT2006 (DH), WT2018 (SF), and WT2019 (HK) VLPs, were minimal, with EC_50_ values equal to or less than the limit of detection (= 50).

**Fig 5:**
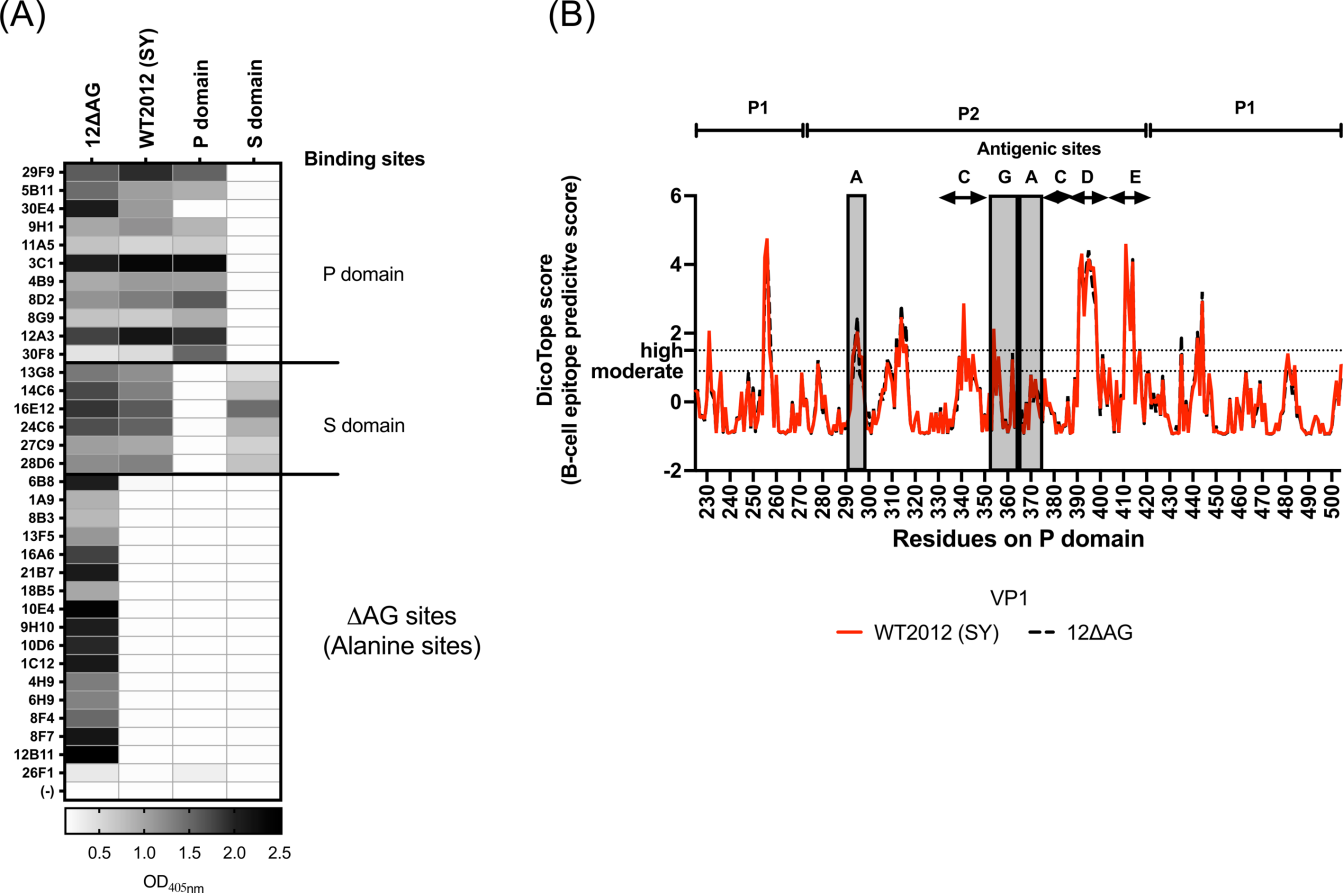
Follow-up analyses of antibodies and 12∆AG VLPs. (**A**) ELISA using the supernatant from 34 parental hybridoma clones obtained after the initial fusion step from mice immunized with 12∆AG VLPs. The heatmap shows OD_405nm_ values in ELISA, using 1:2 dilution of the supernatant from hybridoma clones and 0.5 µg/mL of proteins or VLPs. (**B**) The line graph presents site-by-site B-cell epitope prediction scores generated using DiscoTope-3.0- and AlphaFold-predicted three-dimensional structures of wild-type WT2012 (SY) (red solid line) and 12∆AG mutant VP1 (black dashed lines). The horizontal dashed lines indicate suggested thresholds of high (= 1.5) or moderate (= 0.9) confidence of epitope prediction (33).

We then reevaluated the supernatant of the entire original panel of parental hybridoma clones (*n* = 34, Fig. 5A) and found that while over half of WT2012 (SY) VLP-positive clones mapped to the P domain and were cross-reactive to different variants (Fig. 2), a significant number of clones (16 out of 34) were positive only to the immunogen, 12∆AG VLPs, but negative to WT2012 (SY). This indicates that half of the antibodies mapped to epitopes that included the alanine-mutated sites. The *in silico* analyses using B-cell epitope prediction tools confirmed no major differences in B-cell epitope predictive scores on antigenic sites A and G between WT2012 (SY) and the 12∆AG mutant (Fig. 5B). Antigenic sites A and G still presented strong immunodominance despite the residues on both sites being mutated to alanine.

## DISCUSSION

Various strategies have been proposed to develop universal vaccines against rapidly evolving viruses, including amino acid resurfacing, structural deletion, consensus or mosaic of variable residues, and masking of variable epitopes (34–41). For norovirus vaccines, chimeric and consensus VLPs of different GII.4 variants were developed and confirmed to induce broad blockade responses against GII.4 noroviruses that circulated in different time periods (42, 43). The consensus VLP vaccine candidate has been subjected to clinical trials and demonstrated potential for cross-genotype protection; however, vaccine efficacy was modest in adults (61.8%) and few cross-neutralizing responses were observed (44, 45). In this study, we resurfaced immunodominant epitopes from the variable antigenic sites A and G of GII.4 norovirus with the goal of shifting immune responses toward conserved regions and enhancing cross-immunity.

The alanine-resurfaced mutant VLPs elicited multiple antibodies that mapped to the P domain and reacted with 14 historical GII.4 variants. HBGA-blockade assays further confirmed elicitation of four cross-blocking mAbs. Notably, despite eliciting multiple cross-blockade antibodies, resurfacing of antigenic sites A and G using alanine did not induce sufficient cross-blocking antibodies at the serum level. The observation that a significant number of antibodies were mapped to the resurfaced area, coupled with the lack of significant differences in B-cell epitope predictive scores before and after the resurfacing, suggests that the immunogenicity of antigenic sites A and G remained high despite the introduced mutations. Because of the hydrophobic nature of alanine, further studies using less immunogenic amino acids are warranted in the design of new immunogens.

An additional factor that could contribute to the low blockade titers of the sera induced by alanine-resurfaced mutant VLPs is that the potency of blockade antibodies mapping to the antigenic sites C, D, and other conserved areas of the P domain is not strong (blockade EC_50_ titers ≥ 1.18 µg/mL). Previous studies reported strong potency (low blockade EC_50_ titers, ≤1 µg/mL) of those mapping on antigenic sites A and/or G (18, 46–48), but lower potency for those mapping to other regions (18, 49). Moreover, recent studies suggested that most of the cross-reactivity of WT2012 (SY) sera against WT2006 (DH) and WT2019 (HK) is explained by the presence of cross-blockade antibodies mapping on antigenic site A (50) or G (13). This also explains the low cross-blockade titers of mouse sera elicited against alanine-resurfaced mutant VLPs (Fig. 4). Thus, despite eliciting the cross-blockade antibodies, the potency of blockade antibodies mapping to the antigenic sites C, D, and other conserved areas of the P domain is still modest in producing functional responses at a polyclonal serum level.

Lastly, surface topology, structural stability, and/or conformation of epitopes might be indirectly altered by resurfacing 14 residues on the antigenic sites A and G. A previous study showed that resurfacing of antigenic site A alone could abolish the binding of VLPs to the HBGA molecules (18), although the resurfaced regions are not directly interacting with the HBGAs (51, 52). While ELISA indicated only a minor impact on antibody binding to the alanine-resurfaced VLPs (Fig. 1E), potential structural changes might have affected the immunogenicity, specificity of antibodies to the wild-type viruses, and the potency of cross-blockade antibodies.

In conclusion, we report here that alanine-resurfaced mutant GII.4 VLPs could elicit cross-blockade antibodies, yet multiple factors hindered the inducing of potent blocking responses at the serum level upon immunization with these mutant VLPs. Further studies are required to overcome (i) the low immunogenicity of conserved epitopes and (ii) the low HBGA-blocking potency of most antibodies mapping outside the antigenic sites A and G. Recently, broadly neutralizing antibodies targeting antigenic site A have been identified in people infected with norovirus (53), and this finding could also open another direction of development of universal norovirus vaccines. The presence of such antibodies has been confirmed only in few individuals (54), so future studies are needed to address the mechanisms leading to the generation of broadly neutralizing antibodies to variable and conserved regions of the norovirus capsid protein.

## MATERIALS AND METHODS

### Genome sequence profiling

To summarize genetic diversity among different GII.4 variants, representative VP1-coding sequences from each variant were aligned using MUSCLE, as implemented in MEGA v11 (55). These sequences correspond to viruses that were used in our previous studies to develop wild-type VLPs (13, 15). Evolutionary relationships among variants were presented using a phylogenetic tree using the maximum-likelihood method, as implemented in MEGA v11 (55). The structural positions of antigenic sites and mutations of interest were rendered using UCSF Chimera v1.18 and the VP1 structural data deposited in RCSB Protein Data Bank (PDB ID: 7K6V).

### VLP production

The VP1-encoding sequences of wild-type GII.4 viruses, including WT1974 [CHDC5191/United States/1974; JX023286], WT1987 (CA) [MD145-12/United States/1987; AY032605], WT1996 (GR) [Arizona/United States/1996; AF080556], WT2004 (FH) [MD-2004/United States/2004; DQ658413], WT2004 (HT) [Cumberland/United States/2004; EU078414], WT2005 (SA) [Sakai/Japan/2005; AB220922], WT2006 (YE) [Yerseke38/Netherlands/2006; EF126963], WT2006 (DH) [Den Haag89/Netherlands/2006; EF126965], WT2007 (OS) [Osaka/Japan/2007; AB434770], WT2008 (AP) [Iwate4/Japan/2008; AB541274], WT2010 (NO) [Virginia/Unites States/2010; KX353958], WT2012 (SY) [RockvilleD1/United States/2012; KY424328], WT2018 (SF) [561/Gabon/2018; MW506849], and WT2019 (HK) [CUHK-NS-2200/Hong Kong/2019; MN400355], were synthesized and cloned into pFastBac1 vectors (GenScript) and transformed into MAXEfficiency DH10Bac cells (Gibco) using Bac-to-Bac Baculovirus Expression System (Gibco), as described previously (13, 15). The transformed bacmid was transfected into Sf9 cells (ATCC) using Grace’s Insect Medium (Gibco) and Cellfectin II (Gibco) to produce VP1 proteins, which self-assembled to form VLPs. The produced VLPs were purified using a 25% sucrose cushion (4 hours, 24,000 rpm at 4°C) and cesium chloride gradient (18 hours, 48,000 rpm at 15°C). Expression of the VP1 protein was confirmed by SDS-PAGE using Mini-PROTEAN TGX Stain-Free Precast Gels (BioRad) and Western blot with norovirus VP1-specific cross-reactive mAb, 30A11 (22). Integrity of VLPs was confirmed by negative stain electron microscopy. The 12∆AG mutant with mutations of entire residues on the antigenic sites A and G to alanine was designed in MEGA v11. The VP1 with mutations was synthesized, cloned, and subjected to the Bac-to-Bac Expression system for producing 12∆AG mutant VLPs, as described above. Similarly, antigenic site-swapped mutant VLPs (i.e., individual antigenic sites C or D were swapped between Sydney 2012 and Farmington Hills 2002 variants) were designed and produced, as described previously (18) (Fig. S3).

### S and P domain protein production

The S- and P domain-encoding sequences of WT2012 (SY) with His tag were previously synthesized and cloned into pET28a-MBP vectors (GenScript) (18). The proteins were expressed in *E. coli* and purified with the MBP column, followed by TEV protease cleavage and additional purification with the Ni column and Superdex 200 column (GenScript). Expression of the S and P monomers was confirmed with SDS-PAGE and liquid mass-spectroscopy (Fig. S4).

### Murine mAb production

Initially, six female mice (3 BALB/c and 3 C57BL/6N, Beijing Vital River Laboratory Animal Technology Co., Ltd., strain codes #211 and #219, respectively), aged 6 to 8 weeks, were inoculated with 12∆AG VLPs to measure antibody responses using test serum collected at the 5th week after primary immunization with Complete Freund’s Adjuvant and two boosters with Freund’s Incomplete Adjuvant at 2nd and 4th weeks (GenScript). Based on the preliminary ELISA analyses with the test serum, two C57BL/6 N mice with the highest avidity to WT2012 VLPs were selected for isolation of mAbs. From the two selected mice, B cells were isolated from their spleen and fused with myeloma cells (SP2/0) to form hybridomas 4 days after the final boost at the 7–8^th^ weeks. Hybridomas were diluted to obtain individual clones, and supernatants of the parental clones were screened by ELISA with 12∆AG VLPs, followed by secondary screening with WT2012 (SY) and WT2004 (FH) VLPs, and S and P domain proteins. Eleven clones that were positive to VLPs and P domain were further propagated to produce ten mAbs (one parental clone was not successfully subcloned), which were purified using protein A (GenScript).

### Mouse serum production

Hyperimmune sera against wild-type or 12∆AG mutant VLPs were produced in four female, 5–6 weeks old, BALB/c mice (Charles River Laboratories) following a schedule of intramuscular VLP immunization (40 µg VLPs/dose) and two boosts at the 4th and 8th week adjuvanted with alum (Alhydrogel adjuvant 2%, CRODA), as described previously (15). Cardiac terminal bleed was performed on week 10. Serum was separated from whole blood using BD Microtainer separator tubes (BD Diagnostics).

### Western blot

To characterize the purified mAbs, SDS-PAGE and Western blot analysis of mAbs were performed, as described previously (18). WT2012 (SY) VLPs (100 µg/mL) were mixed with Laemmli Sample Buffer (BioRad) and 5% 2-mercaptoethanol (BioRad) and incubated at 95°C for 5 min to linearize the VP1 proteins. The 10 µL of denatured VLPs was loaded on Mini-PROTEAN Precast Gel (BioRad) and run for 30 min at 200V and was transferred to a nitrocellulose membrane. The membrane was blocked overnight at 4°C with 2% blocking buffer (BioRad) and incubated with 1 µg/mL of individual mAbs for 1 hour at room temperature. The membrane was washed three times with 1 × PBS, 0.1% Tween 20 and incubated with goat anti-mouse IgG-HRP (SeraCare) for 1 hour at room temperature. Binding of mAbs to the denatured VP1 protein was visualized using SuperSignal West Pico PLUS Chemiluminescent Substrate (Thermo Scientific) and ChemiDoc Touch Imaging System (BioRad).

### ELISA

To profile the binding pattern of purified mAbs and overall IgG titers in the serum, ELISA with S domain, P domain, and/or VLPs was performed as described previously (18). Briefly, 96-well ‘‘U’’ bottom microtiter plates were coated with 100 µL of 0.5 µg/mL proteins or VLPs in 1 × PBS (pH 7.4) overnight at 4°C and blocked for 1 hour at room temperature with 5% blocking buffer (BioRad). A panel of mAbs (10 µg/mL each or 10-fold serial dilution starting at 100 µg/mL) or serum (10-fold serial dilution starting at 1:100 dilution) was plated in duplicate and incubated with antigens (0.5 µg/mL each) for 2 hours at room temperature. Plates were washed three times with 1 × PBS, 0.1% Tween 20. Binding antibodies on VLPs were incubated with HRP-conjugated goat anti-mouse IgG (SeraCare) for 1 hour at room temperature. After three washes, ABTS (SeraCare) was added and incubated for 10 min at room temperature until the reaction was quenched by 1% SDS. Binding signals were measured as OD_405nm_ using a SPECTROstar Nano plate reader. The EC_50_ values were calculated from the normalized OD_405nm_ curves using GraphPad Prism v10. Statistical tests were conducted using GraphPad Prism v10.

### HBGA-blockade assays

HBGA-blockade assays were conducted using porcine gastric mucin III (PGM III) (Sigma-Aldrich) as a source of HBGA carbohydrates. Briefly, 100 µL of PGM III (10 µg/mL in 1 × PBS, pH 7.4) was coated on 96-well “U” bottom vinyl plates (Costar) overnight at 4°C and blocked for 1 hour at room temperature with 5% blocking buffer (Bio-Rad). Another 96-well “U” bottom vinyl plate was blocked overnight at 4°C and used to mix and pre-incubate antibodies and VLPs. Duplicates of twofold serial dilutions of mAbs (100 µg/mL) or hyperimmune serum (1:100) were mixed with VLPs at 0.5 µg/mL and incubated for 1 hour at 37°C. One hundred microliters of pre-incubated antibody-VLPs mixture was then added to PGM III-coated plates and incubated for an additional 1 hour at 37°C. Pooled sera from guinea pigs immunized with WT2012 (SY) and WT2004 (FH) VLPs were used as primary detection antibodies against VLPs that were bound to the HBGA carbohydrate coated on the plate. Binding (or blocking of binding) of VLPs on the HBGA carbohydrate was determined using goat anti-guinea pig IgG-HRP and ABTS (SeraCare). The OD_405nm_ values were measured using a SPECTROstar Nano plate reader (BMG LABTECH). The EC_50_ values (measured by mAb concentration or reciprocal of serum dilution) were calculated from the normalized OD_405nm_ curves using GraphPad Prism v10. Statistical tests were conducted using R v4.4.1 or GraphPad Prism v10.

### B-cell epitope prediction

The VP1 amino acid sequences of WT2012 (SY) and 12∆AG mutant were submitted to AlphaFold2 (56) implemented in FDA High-performance Integrated Virtual Environment (57) to predict their three-dimensional structures. The predicted structures were then used as input files of DiscoTope-3.0 to estimate site-by-site B-cell epitope prediction scores (33). The prediction scores were visualized using GraphPad Prism v10.

## Data Availability

The viral sequences used to develop the VLPs are available in Materials and Methods. VLPs, sera, and mAbs used in this study are available upon request.

## SUPPLEMENTAL MATERIAL

Supplemental figures jvi.00611-25-s0001.docx Additional data to present ELISA curves, SDS-PAGE gels, and electron microscopy images of the VLPs.

ASM does not own the copyrights to Supplemental Material that may be linked to, or accessed through, an article. The authors have granted ASM a non-exclusive, world-wide license to publish the Supplemental Material files. Please contact the corresponding author directly for reuse.
