# Supplementary material for: Manipulation of immunodominant variable epitopes of norovirus capsid protein elicited cross-blocking antibodies to different GII.4 variants despite the low potency of the polyclonal sera
Source: J Virol. 2025 May 30;99(7):e00611-25. doi: 10.1128/jvi.00611-25 (PMC12282063; doi:10.1128/jvi.00611-25)
Supplement: Supplemental figures — Additional data to present ELISA curves, SDS-PAGE gels, and electron microscopy images of the VLPs. [file jvi.00611-25-s0001.docx]

**Supplementary Materials**

**Figure S1:** ELISA OD_405nm_ curves to show the binding of anti-WT2012 mAbs against WT2012 and the 12∆AG VLPs. The lines indicate best-fit linear regression curves and normalized OD_405nm_ values in duplicate wells at 10-fold dilution of mAbs.

**Figure S2:** HBGA-blockade OD_405nm_ curves to show the blocking of anti-12∆AG mAbs against different GII.4 variants VLPs. The lines indicate best-fit linear regression curves and normalized OD_405nm_ values in duplicate wells at 2-fold dilution of mAbs.

**Figure S3:** The electron microscopy images of antigenic sites C and D-swapped mutant VLPs.

**Figure S4:** The SDS-PAGE gel images of expressed P domain and S domain.
